# An Integrative Pan-Cancer Analysis of Kinesin Family Member C1 (KIFC1) in Human Tumors

**DOI:** 10.3390/biomedicines10030637

**Published:** 2022-03-10

**Authors:** Hao Wu, Yingjuan Duan, Siming Gong, Qiang Zhu, Xuanyou Liu, Zhenguo Liu

**Affiliations:** 1 Center for Precision Medicine and Division of Cardiovascular Medicine, University of Missouri School of Medicine, Columbia, MO 65212, USA; hw5mg@missouri.edu (H.W.); zhuqia@health.missouri.edu (Q.Z.); liuxua@health.missouri.edu (X.L.); 2 Faculty of Chemistry and Mineralogy, University of Leipzig, 04103 Leipzig, Germany; yingjuan.duan@studserv.uni-leipzig.de; 3 Institute of Anatomy, University of Leipzig, 04103 Leipzig, Germany; siming.gong@studserv.uni-leipzig.de

**Keywords:** KIFC1, pan cancer, prognosis, MDSCs, immunotherapy

## Abstract

Kinesin family member C1 (KIFC1) is a minus-end-directed motor protein that is critically involved in microtubule crosslinking and spindle formation. KIFC1 is essential for supernumerary centrosomes, and it is associated with the initiation and progression of cancers. In the present study, we initially reviewed the The Cancer Genome Atlas database and observed that *KIFC1* is abundantly expressed in most types of tumors. We then analyzed the gene alteration profiles, protein expressions, prognoses, and immune reactivities of KIFC1 in more than 10,000 samples from several well-established databases. In addition, we conducted a gene set enrichment analysis to investigate the potential mechanisms for the roles of *KIFC1* in carcinogenesis. The pan-cancer analysis of KIFC1 demonstrates significant statistical correlations of the KIFC1 expression with the clinical prognoses, the oncogenic signature gene sets, the myeloid-derived suppressor cell infiltration, the ImmunoScore, the immune checkpoints, the microsatellite instabilities, and the tumor mutational burdens across multiple tumors. These data may provide important information on the understanding of the role and mechanisms of KIFC1 in carcinogenesis and immunotherapy, as well as on the clinical progression of a variety of cancers.

## 1. Introduction

The kinesin family member C1 (KIFC1) gene was first identified within a segment centromeric to the class II gene region of the human major histocompatibility complex in 1992 [1]. It is abundantly expressed in bone marrow, lymph nodes, and many other tissues in the gastrointestinal system, the skin, the spleen, the testis, and the bladder [2]. KIFC1, which is a microtubule-binding protein of the kinesin-14 family, is a minus-end-directed motor protein that is critically involved in microtubule crosslinking and spindle assembly in mammalian cells [3,4]. KIFC1 is essential for supernumerary centrosomes, which is known as “centrosome amplification”, and it promotes the multipolar spindle formation in cells. Recent data suggest that KIFC1 may also play an important role in vesicular and organelle trafficking and spermiogenesis, as well as in neuronal migration [5,6,7].

KIFC1 has been shown to drive chromosome segregation errors and aneuploidy, which results in the initiation and/or acceleration of carcinogenesis [8,9,10,11]. Indeed, recent studies have shown that KIFC1 is highly expressed in prostate cancer, hepatocellular carcinoma, serous ovarian adenocarcinomas, non-small cell lung cancer, renal cell carcinoma, triple-negative breast cancer, and that it is associated with poor prognoses [12,13,14,15,16,17]. KIFC1 is also associated with docetaxel resistance in breast cancer and prostate cancer [18,19]. Some studies suggest that KIFC1 may be involved in tumor recurrence [13,20]. The depletion of KIFC1 leads to dramatic increases in multipolar anaphases, and to the selective cell death of the cancer cells with centrosome amplification [21], which provide a potential new target for effective cancer therapy. In fact, KIFC1 inhibitors (e.g., AZ82, CW069, and SR31527) have been identified and being studied for potential therapy of different forms of cancers [22,23,24].

The underlying mechanism(s) for the roles of KIFC1 in carcinogenesis, and for the drug resistance of various tumors, have not been well defined. We reviewed The Cancer Genome Atlas (TCGA) database and found that KIFC1 is abundantly expressed in the majority of tumors of diverse origins. Thus, a pan-cancer analysis of KIFC1 may provide new insights into the molecular mechanisms for tumor occurrence, recurrence, and immunotherapy. In the present study, we analyzed the profiles of the gene alteration, protein expression, prognosis, and immune reactivity of KIFC1 in more than 10,000 samples from several well-established databases. We also conducted a KIFC1-related gene enrichment analysis to investigate the potential mechanisms for the role of KIFC1 in carcinogenesis. The objective of this study was to examine the roles and potential mechanisms of KIFC1 in the development and progression of human tumors.

## 2. Materials and Methods

### 2.1. Genetic Alteration Analysis

The genetic alteration features of *KIFC1* were obtained from the “TCGA PanCancer Atlas Studies” module of the cBioPortal website (https://www.cbioportal.org/, accessed on 2 January 2022) [25]. The information on the tumor-entity summaries, the alteration frequencies, and the copy number alterations (CNAs) is shown in the “Cancer Types Summary” module. The diagram of the *KIFC1* alteration sites, which includes the alteration types and the case numbers, was generated from the “mutations” module.

### 2.2. Gene Expression Analysis

“*KIFC1*” was inputted into the “Gene_DE” module of the Tumor Immune Estimation Resource version2 (TIMER2) website (http://timer.cistrome.org/, accessed on 2 January 2022). The difference between the *KIFC1* expression in the tumor tissues and the adjacent normal tissues in different tumors, or specific tumor subtypes, was evaluated in the TCGA database. The adjacent normal tissues were considered as the controls. If there was no control group for the tumors in the TCGA database, the Genotype-Tissue Expression (GTEx) database was used for the corresponding normal tissues as the controls. The difference in the *KIFC1* expression between certain tumor tissues and normal tissues was further examined with the “Box Plot” module of the Gene Expression Profiling Interactive Analysis, version2 (GEPIA2) website (http://gepia2.cancer-pku.cn/, accessed on 2 January 2022) [26]. Additionally, the violin plots of the *KIFC1* expression in different pathological stages of tumors in TCGA database were created via the “Pathological Stage Plot” module of the GEPIA2. The UALCAN (http://ualcan.path.uab.edu/analysis-prot.html, accessed on 4 January 2022) was used to conduct the KIFC1 protein expression analysis in the Clinical Proteomic Tumor Analysis Consortium (CPTAC) Confirmatory/Discovery database [27]. The Human Protein Atlas (HPA) database (https://www.proteinatlas.org/, accessed on 4 January 2022) was used to obtain the *KIFC1* RNA expression levels in the normal tissues, and the immunohistochemical staining images of the KIFC1 protein in human tumors and normal tissues.

### 2.3. Survival Analysis

The “Survival Map” module of the GEPIA2 was utilized to obtain the overall survival (OS) and disease-free survival (DFS) heatmap data of KIFC1 across all of the tumors in the TCGA cohort. The log-rank test was applied to the hypothesis test, and the survival plots were also obtained through the “Survival Analysis” module of the GEPIA2.

### 2.4. Protein–Protein Interactions of KIFC1 and Similar Genes in Pan Cancer

Using the STRING tool (https://string-db.org/, accessed on 5 January 2022), a protein–protein interaction (PPI) analysis of KIFC1 was performed with 50 available experimentally determined proteins that interacted with KIFC1, and that were visualized in the PPI network. On the basis of the tumor data from the TCGA cohort, the top 100 *KIFC1*-correlated targeting genes were obtained from the “Similar Genes Detection” module of the GEPIA2. The “correlation analysis” module of the GEPIA2 was applied in order to conduct a pairwise gene Pearson’s correlation analysis of *KIFC1* and the top five selected genes. In addition, a Spearman’s correlation test was performed for the top five selected genes by using the “Gene_Corr” module of the TIMER2 website to obtain the heatmap. 

By using the Venn Diagram (http://bioinformatics.psb.ugent.be/webtools/Venn/, accessed on 5 January 2022) [28], we conducted an intersection analysis to compare the *KIFC1*-interacted and *KIFC1*-correlated genes. In addition, after combining the two sets of data, the R package, “clusterProfiler”, in the R software (Version 4.1.1, R Foundation for Statistical Computing, Vienna, Austria) was used to perform the Kyoto Encyclopedia of Genes and Genomes (KEGG) pathway analysis and the Gene Ontology (GO) enrichment analysis, which included the biological processes, the molecular functions, and the cellular components.

### 2.5. Gene Set Enrichment Analysis of KIFC1 in Pan Cancer

To explore the biological and oncogenic signaling pathways, a gene set enrichment analysis (GSEA) was performed on high- and low-expression groups on the basis of the mean expression values of KIFC1 in 33 tumors of the TCGA dataset. The R package, “clusterProfiler”, in the R software, was used to perform the MSigDB H (hallmark gene sets) and C6 (oncogenic signature gene sets) enrichment analyses [29]. The gene sets with |NES| > 1, p.adjust < 0.05, and FDR < 0.25, were considered to be enrichment significant.

### 2.6. Immune Reactivity Analysis

To evaluate the association between the *KIFC1* expression and the immune infiltration of all of the tumors in the TCGA database, “*KIFC1*” was inputted into the “gene expression” module, while “myeloid-derived suppressor cells” (MDSCs) and “cancer-associated fibroblasts” (CAFs) were inputted the into the “immune infiltrates” module of the TIMER2 website in order to obtain a heatmap and the scatter plots. The SangerBox (http://sangerbox.com/, accessed on 5 January 2022) online platform was used to calculate the Stromal, Immune, and ESTIMATE scores [30]. In addition, through SangerBox, the relationships between the KIFC1 expression and the various immune checkpoints were explored. The correlations between the *KIFC1* expression and the microsatellite instability (MSI) and the tumor mutational burden (TMB) in different tumors of the TCGA database were also analyzed by using SangerBox.

### 2.7. Statistical Analysis

The gene expression data from the TCGA and GTEx databases were analyzed by using the Wilcoxon test. The protein expression data from the UALCAN dataset were analyzed by using the Student’s *t*-test. The survival data from the GEPIA2 database were analyzed by using the log-rank test. The R package, “clusterProfiler”, in the R software (Version 4.1.1, R Foundation for Statistical Computing, Vienna, Austria) was used to perform the KEGG pathway, GO, MSigDB H, and C6 enrichment analyses. The correlation analysis was evaluated in the TIMER2 database by using a purity-adjusted Spearman’s rho. The correlation analysis of the ImmuneScore, the StromalScore, the ESTIMATEScore, the immune checkpoints, the MSI, and the TMB used Pearson’s correlation coefficients. The value, *p* < 0.05, was considered statistically significant.

## 3. Results

### 3.1. Genetic Alteration of KIFC1 in Tumors

**Figure 1:**
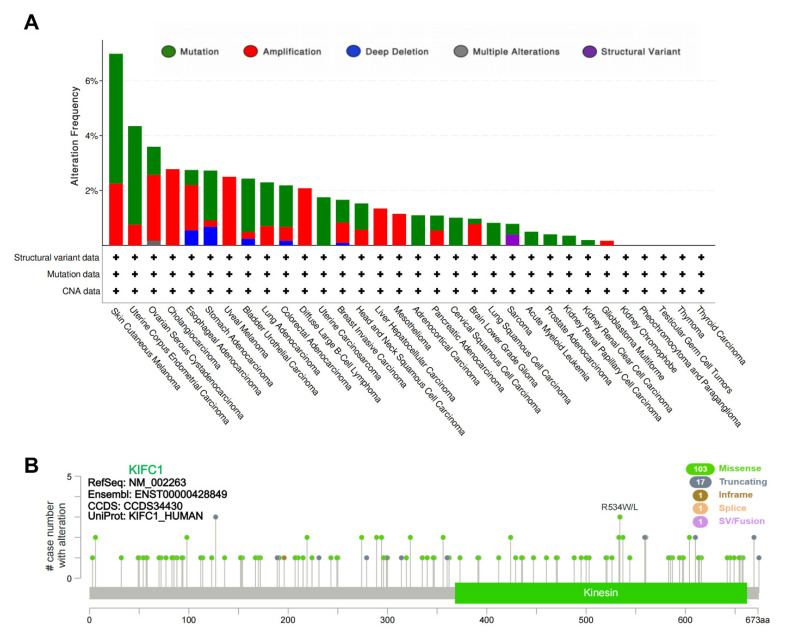
Genetic alterations of *KIFC1* in different tumors of TCGA database. (**A**) Alteration frequencies with mutation types. (**B**) The mutation sites and case numbers of *KIFC1* genetic alterations. CNA: copy number alteration; SV: structural variation.

The following tumor entities from the TCGA database were included in this study: ACC, BLCA, BRCA, COAD, CHOL, CESC, DLBC, ESCA, GBM, HNSC, KICH, KIRC, KIRP, LAML, LGG, LIHC, LUAD, LUSC, MESO, OV, PAAD, PCPG, PRAD, READ, SARC, SKCM, STAD, TGCT, THCA, THYM, UCEC, UCS, and UVM (abbreviations and acronyms are listed in Table S1). The *KIFC1* genetic alteration profiles of the tumors in the TCGA database show that 1.7% of the enrolled patients had genetic alterations (predominantly missense mutations and amplifications), and the patients with SKCM had the highest frequency (6.98%) of *KIFC1* genetic alterations (Figure 1A). As is shown in Figure 1B, missense was the most common type of mutation, which was followed by the truncating mutation. Of note, all of these somatic mutations were classified as “variants of uncertain significance”.

### 3.2. Gene Expression Analysis Data

**Figure 2:**
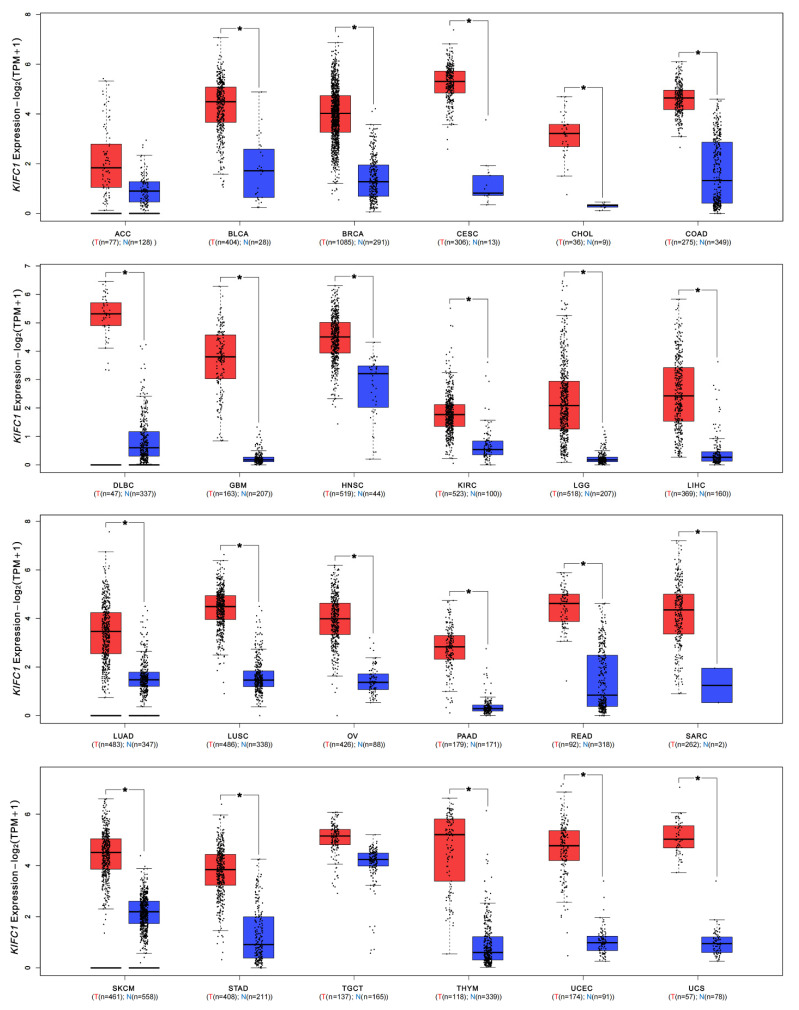
mRNA expression levels of KIFC1 in different tumors. The *KIFC1* expression levels of different cancers in the TCGA database compared with normal tissues in GTEx database; Log_2_ (TPM + 1) transformed the expression data for plotting. * *p* < 0.05, in Wilcoxon test. TPM: transcripts per million; N and T: normal and tumor tissues.

**Figure 3:**
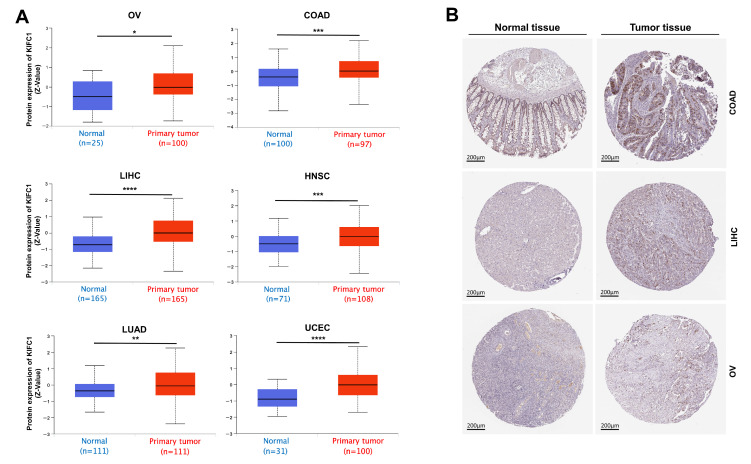
Protein expression of KIFC1 in different tumors. (**A**) The KIFC1 total protein expression between normal tissue and primary tumor tissue, according to the CPTAC database; Zvalues represent standard deviations from the median across samples for a given cancer type. * *p* < 0.05, ** *p* < 0.01, *** *p* < 0.001, **** *p* < 0.0001, in Student’s *t*-test. TPM: transcripts per million. (**B**) KIFC1 immunohistochemistry staining images in human COAD, LIHC, and OV, compared with normal tissues from HPA database.

The expression patterns of *KIFC1* in different normal tissues, which are based on the consensus datasets of the HPA and GTEx databases, are shown in Figure S1. *KIFC1* was abundantly expressed in bone marrow and lymphoid tissue, with high RNA levels. An analysis of the expression profiles of *KIFC1* in different tumor tissues and normal tissues in the consensus databases of TCGA and GTEx shows that the expression levels of *KIFC1* were significantly higher in tumor tissues than in normal tissues across different types of cancers, such as BLCA, BRCA, CESC, CHOL, COAD, DLBC, ESCA, GBM, HNSC, KIRC, KIRP, LGG, LIHC, LUAD, LUSC, OV, PAAD, PCPG, PRAD, READ, SARC, SKCM, STAD, THCA, THYM, UCEC, and UCS (Figure 2 and Figure S2). In addition, it was found that the expression levels of *KIFC1* were significantly related to the pathological stages of BRCA, KICH, KIRC, KIRP, LIHC, and LUAD (Figure S3). In the CPTAC database, the total KIFC1 protein expressions were higher in primary cancers than in normal tissues for OV, COAD, UCEC, LIHC, HNSC, and LUAD (Figure 3A). The immunohistochemistry staining images from the HPA database show that positive KIFC1 staining was present in the tissues of COAD, LIHC, and OV, and not in the normal tissues (Figure 3B).

### 3.3. Survival Analysis Data

**Figure 4:**
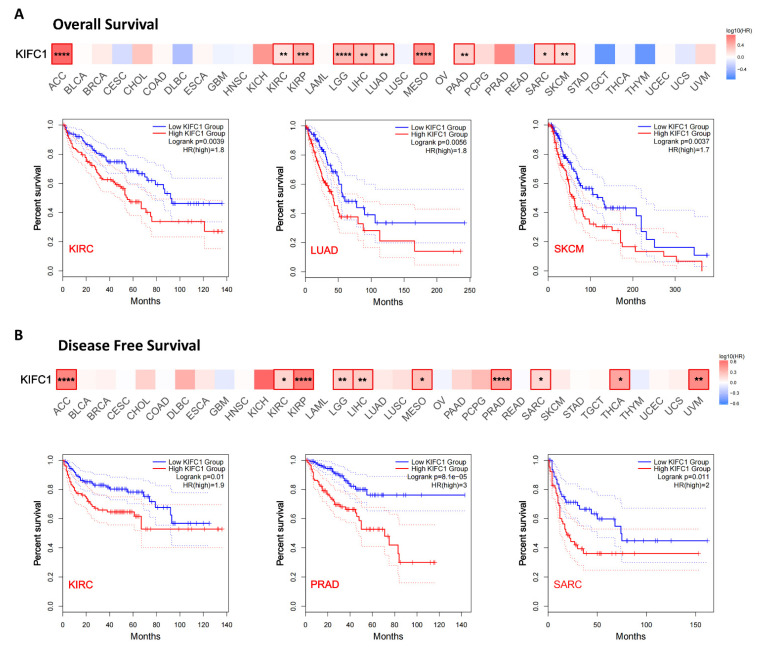
Relations between KIFC1 expression and survival prognoses of different tumors in TCGA database are shown in survival maps and with Kaplan–Meier curves. GEPIA2 tool was used to obtain (**A**) the overall survival, and (**B**) disease-free survival analyses. High-cutoff (50%) and low-cutoff (50%) values were used as the expression thresholds for separating the high-expression and low-expression cohorts. * *p* < 0.05, ** *p* < 0.01, *** *p* < 0.001, **** *p* < 0.0001, in log-rank test. HR: hazard ratio.

On the basis of the levels of the *KIFC1* expression in the tumors, the correlation between the *KIFC1* expression and the prognoses of the tumors was explored in the TCGA database. It was found that increased levels of *KIFC1* expression were significantly associated with poor OSs of ACC [HR, 6; *p* < 0.0001]; KIRC [HR, 1.5; *p* = 0.014]; KIRP [HR, 2.5; *p* = 0.0036]; LGG [HR, 2.3; *p* < 0.0001]; LIHC [HR, 2.2; *p* < 0.0001]; LUAD [HR, 1.5; *p* = 0.0077]; MESO [HR, 3.4; *p* < 0.0001]; PAAD [HR, 1.8; *p* = 0.0059]; SARC [HR, 1.7; *p* = 0.011]; and SKCM [HR, 1.5; *p* = 0.0029] (Figure 4A). High expression levels of *KIFC1* were also significantly associated with poor DFSs of ACC [HR, 3.3; *p* = 0.0006]; KIRC [HR, 1.6; *p* = 0.015]; KIRP [HR, 3.6; *p* < 0.0001]; LGG [HR, 1.6; *p* = 0.0025]; LIHC [HR, 1.7; *p* = 0.0006]; MESO [HR, 1.9; *p* = 0.031]; PRAD [HR, 2.5; *p* < 0.0001]; SARC [HR, 1.5; *p* = 0.02]; THCA [HR, 2.5; *p* = 0.003]; and UVM [HR, 3.2; *p* = 0.022] (Figure 4B).

### 3.4. Protein–Protein Interactions of KIFC1 and Similar Genes in Pan Cancer

**Figure 5:**
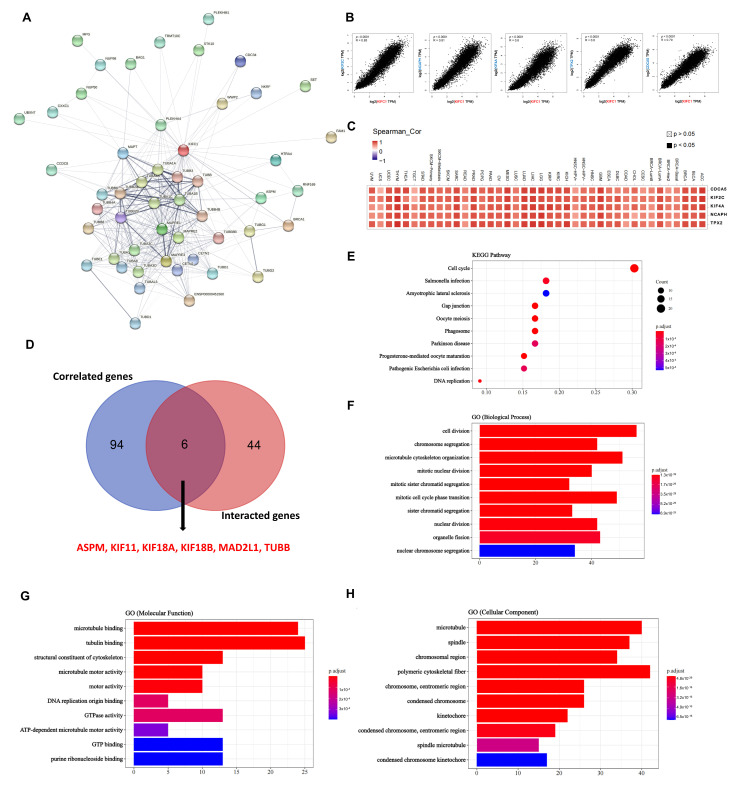
KIFC1-related gene network, KEGG pathway analysis, and GO enrichment analysis. (**A**) A total of 50 available experimentally determined KIFC1-interacted proteins using STRING tool. (**B**) Top 100 *KIFC1*-correlated genes in TCGA database and selected targeting genes, including *CDCA5*, *KIF2C*, *KIF4A*, *NCAPH*, and *TPX2*, in Pearson’s correlation coefficients. (**C**) The corresponding heatmaps of the detailed tumor types, in purity-adjusted partial Spearman’s rho values. (**D**) An intersection analysis of the KIFC1-interacted and *KIFC1*-correlated genes. (**E**) KEGG pathway analysis based on the KIFC1-interacted and *KIFC1*-correlated genes. (**F**–**H**) The bar plots of GO enrichment analysis of biological processes, molecular functions, and cellular components. Adjusted *p*-values were obtained from multiple hypotheses test using the Benjamini–Hochberg procedure; *p*.adjust < 0.05 was considered statistically significant (**E**–**H**).

In order to further investigate the potential mechanism of *KIFC1* in carcinogenesis, we conducted a series of pathway enrichment analyses for the proteins that interacted with KIFC1, and for the genes that correlated with *KIFC1* on the basis of the STRING tool and the GEPIA2. A total of 50 proteins that experimentally interacted with KIFC1 were shown in the PPI network (Figure 5A). In addition, the top 100 *KIFC1*-correlated genes (Table S2) were obtained, among which the top five genes were: kinesin family member 2C (*KIF2C*) (R = 0.85); non-SMC condensin I complex subunit H (*NCAPH*) (R = 0.81); kinesin family member 4A (*KIF4A*) (R = 0.8); targeting protein for Xklp2 (*TPX2*) (R = 0.8); and cell division cycle associated 5 (*CDCA5*) (R = 0.79) (Figure 5B). The corresponding heatmap data show significant and positive correlations between *KIFC1* and the top five genes in all of the tumor types in the TCGA database (Figure 5C). An intersection analysis of the genes that directly interacted with or related to KIFC1 identified two genes, namely, assembly factor for spindle microtubules (*ASPM*), and tubulin beta class I (*TUBB*) (Figure 5D). By using a combination of the two datasets, the KEGG pathway analysis indicates that the “cell cycle” and the “DNA replication” might be the potential mechanisms for the effect of *KIFC1* on carcinogenesis (Figure 5E). The GO enrichment analysis further suggests that the genes that directly interacted with or that were related to KIFC1 were mainly related to the biological processes of “cell division” and “chromosome segregation” (Figure 5F), to the molecular functions of “microtubule binding” and “tubulin binding” (Figure 5G), and to the cellular components of the “microtubule” and the “spindle” (Figure 5H).

### 3.5. Gene Set Enrichment Analysis Data

**Figure 6:**
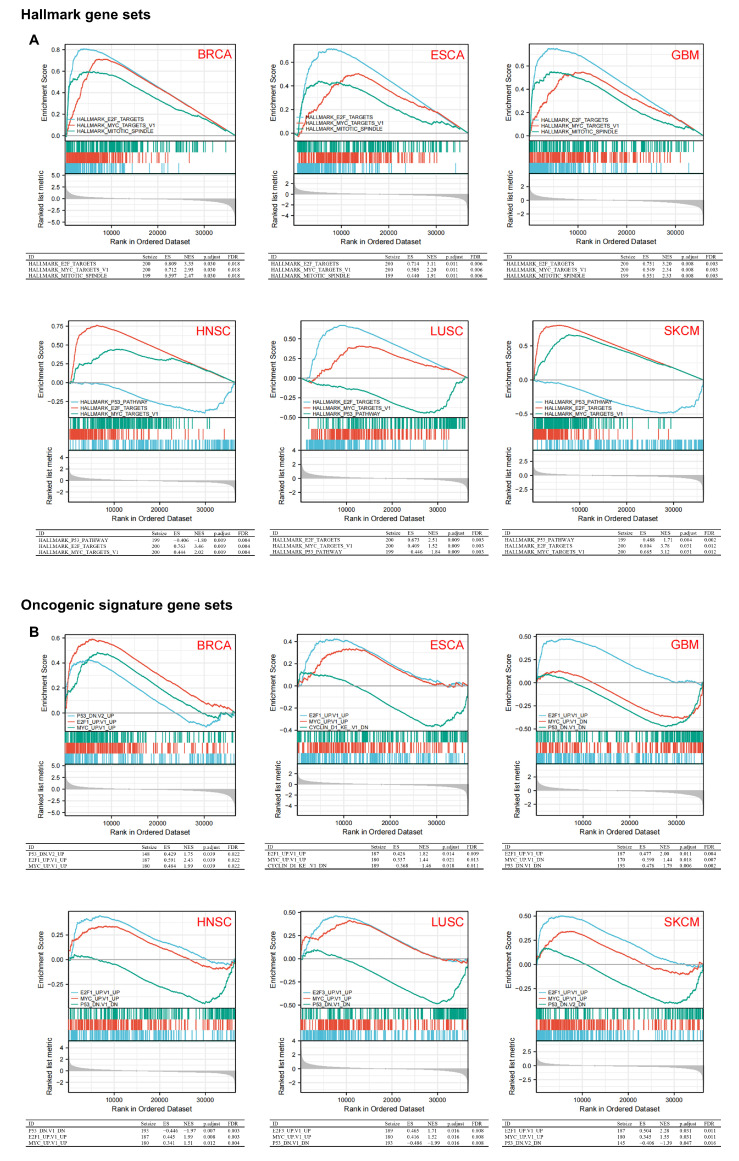
Gene set enrichment analysis of KIFC1. (**A**) Hallmark gene sets enriched in high *KIFC1* expression group. (**B**) Oncogenic signature gene sets enriched in high *KIFC1* expression group. ES: enrichment score; NES: normalized enrichment score; FDR: false discovery rate.

The MSigDB H (hallmark gene sets) and C6 (oncogenic gene sets) databases were analyzed in the present study. The enrichment of H analysis demonstrates that the high expression of *KIFC1* was associated with the genes for mitotic spindle assembly, the cell-cycle-related targets of the E2F transcription factors, the genes regulated by MYC, and the p53 pathways (Figure 6A). The enrichment of C6 analysis shows that the high expression of *KIFC1* was associated with various signature oncogenes, such as *E2F* and *MYC*, whereas the low expression of *KIFC1* was related to the tumor suppression signature gene, *p53* (Figure 6B).

### 3.6. Immune Reactivity Analysis Data

**Figure 7:**
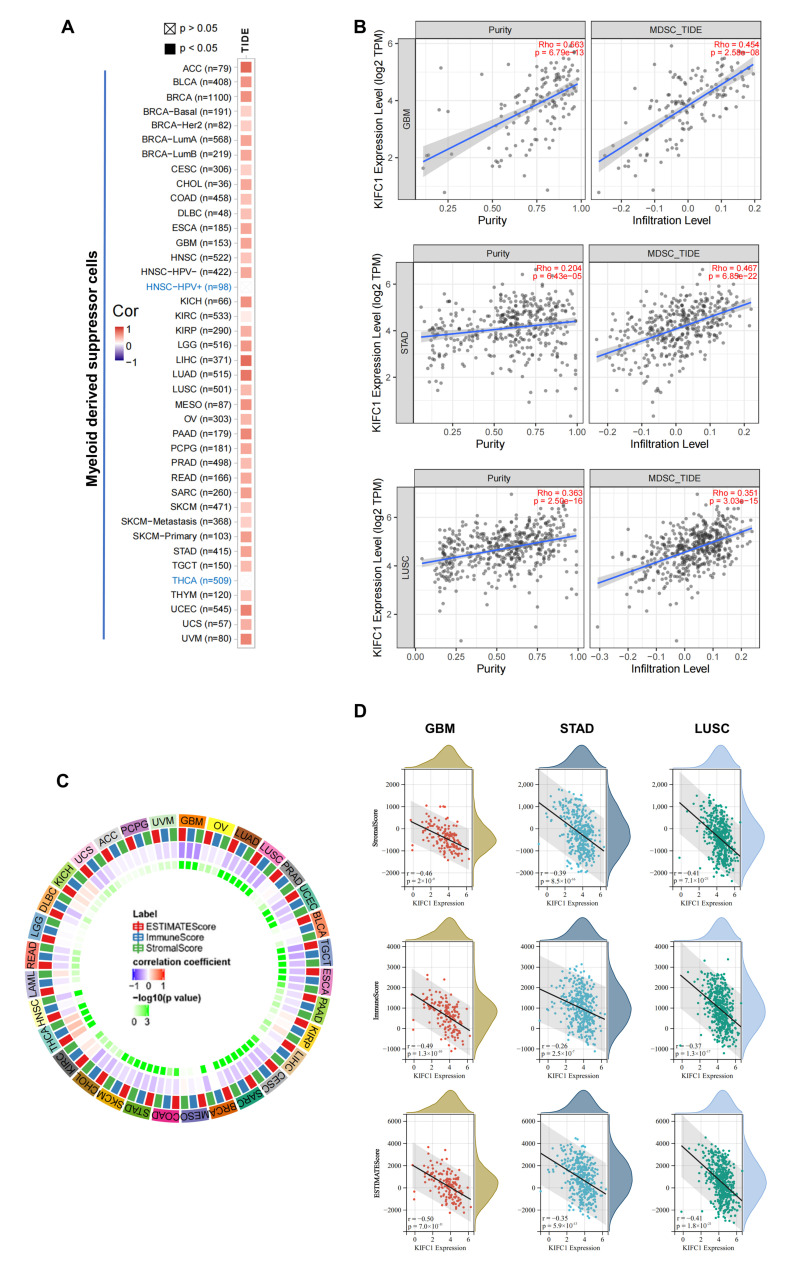
Immune reactivity analysis of KIFC1 in different tumors of TCGA database. (**A**,**B**) Correlation analysis of *KIFC1* expression and immune infiltration of myeloid-derived suppressor cells, in purity-adjusted Spearman’s rho values, with TIDE algorithm. (**C**,**D**) Correlations of ImmuneScore, StromalScore, and ESTIMATEScore with *KIFC1* expression in tumors, in Pearson’s correlation coefficients. TIDE: tumor immune dysfunction and exclusion.

The correlations between the expression of *KIFC1* and the infiltration levels of the MDSCs and CAFs were estimated in the TCGA database. To our surprise, significant and positive correlations between the expression of *KIFC1* and the infiltration of the MDSCs was presented in all of the tumor types, except for HNSC-HPV+ and THCA (Figure 7A). Positive correlations between the *KIFC1* expression and the infiltration estimation values were present for STAD, GBM, and LUSC, as is illustrated in the representative scatter plots (Figure 7B). In addition, statistically significant negative correlations between the *KIFC1* expression and the estimated infiltration values of the CAFs were presented in BRCA, HNSC-HPV+, STAD, and THYM, while positive correlations were noted for KIRP and THCA (Figure S4). By using the SangerBox “Estimate infiltration” module, we calculated the correlations of the ImmuneScore, the StromalScore, and the ESTIMATEScore with the *KIFC1* expression in 32 tumor types on the basis of the TCGA database (Table S3). The *KIFC1* expression in GBM, UCEC, CESC, LUAD, ESCA, SARC, STAD, LUSC, SKCM, OV, and TGCT was significantly and negatively correlated with the ImmuneScore, the StromalScore, and the ESTIMATEScore. On the contrary, the *KIFC1* expression in KIRC and THCA was statistically and positively associated with these three scores (Figure 7C). Negative correlations between the *KIFC* expression and the ImmuneScore, the StromalScore, and the ESTIMATEScore were observed for STAD, GBM, and LUSC, as is shown in the representative scatter plots (Figure 7D).

**Figure 8:**
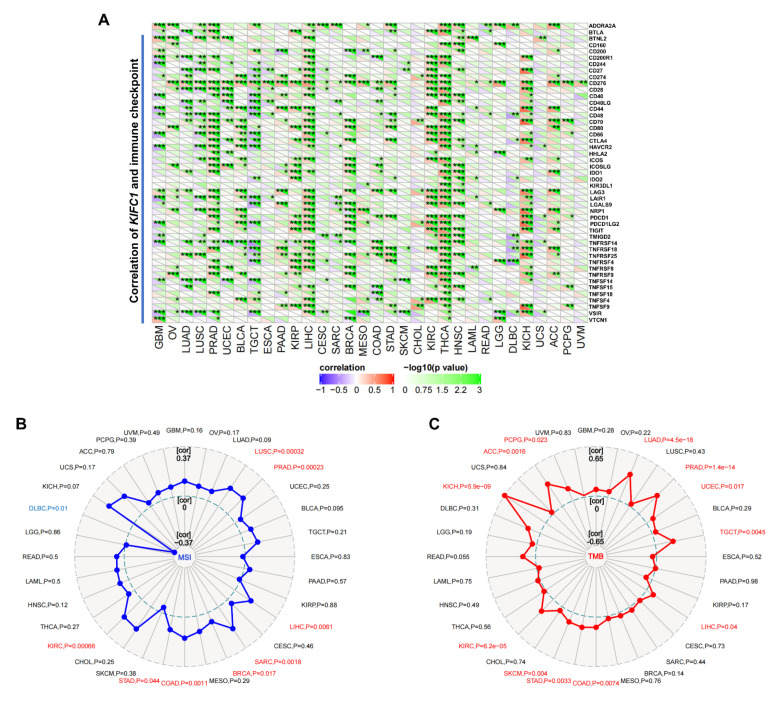
Correlations of KIFC1 expression with immune checkpoints of MSI and TMB in TCGA database. (**A**) Heatmaps represent the color-coded correlations of immune checkpoints and KIFC1 across different tumors. (**B**) Radar chart displays the overlaps between KIFC1 and MSIs. (**C**) Radar chart displays the overlaps between KIFC1 and TMBs. * *p* < 0.05, ** *p* < 0.01, *** *p* < 0.001, in Pearson’s correlation coefficients (**A**–**C**).

The immune checkpoint analysis shows that the expression of *KIFC1* in the PRAD, LIHC, KIRC, THCA, HNSC, and KICH was positively correlated with most of the immune checkpoint genes, especially *CD276*, lymphocyte activating 3 (*LAG3*), and programmed cell death protein 1 (*PD1*) (Figure 8A). An analysis on the relationship between the *KIFC1* expression and the MSIs/TMBs of the tumors in the TCGA database shows that the *KIFC1* expression was significantly and positively correlated with the MSIs in LUSC, PRAD, LIHC, SARC, BRCA, COAD, STAD, and KIRC, whereas it was negatively correlated with the MSIs in DLBC, as is illustrated in the radar chart (Figure 8B). The analysis also demonstrates that the *KIFC1* expression was significantly and positively correlated with the TMBs in LUAD, PRAD, UCEC, TGCT, LIHC, COAD, STAD, SKCM, KIRC, KICH, ACC, and PCPG (Figure 8C).

## 4. Discussion

In the present study, we demonstrate that: (1) The expression level of *KIFC1* was significantly higher in tumor tissues than in normal tissues across most types of tumors in the TCGA cohort; (2) The total KIFC1 protein expression was higher in the primary cancers than in the normal tissues for OV, COAD, UCEC, LIHC, HNSC, and LUAD in the CPTAC database; (3) The high expression of *KIFC1* was significantly associated with poor OSs and DFSs of the various tumors in the TCGA cohort; (4) *KIFC1* was significantly and positively correlated with *KIF2C*, *NCAPH*, *KIF4A*, *TPX2*, and *CDCA5* in all of the tumor types in the TCGA database; (5) The KEGG pathway analysis and the GO enrichment analysis, which were based on the *KIFC1*-interacted and -correlated genes, show that the “cell cycle” and the “DNA replication” might be the mechanisms for the effect of *KIFC1* on carcinogenesis; (6) The high expression of *KIFC1* was significantly associated with the *E2F* and *MYC* signature oncogenes in BRCA, ESCA, GBM, HNSC, LUSC, and SKCM; (7) A significant and positive correlation between the expression of *KIFC1* and the infiltration of MDSCs was present in all of the tumor types, except for HNSC-HPV+ and THCA; (8) The *KIFC1* expression was significantly and negatively correlated with the ImmuneScore, the StromalScore, and the ESTIMATEScore in most of the tumors in the TCGA; (9) The *KIFC1* expression was significantly and positively correlated with the MSI and TMB, and *CD276*, *LAG3*, and the *PD1* immune checkpoints in more than half of the tumors in TCGA.

KIFC1 actively transports bare double-stranded DNA along the cytoskeleton filaments [31], and it is critically involved in centrosome amplification. KIFC1 has also been shown to be associated with the initiation and/or progression of a variety of cancers [10,11]. Several clinical studies have shown that KIFC1 is highly expressed and associated with poor prognoses for STAD, LIHC, BRCA, and OV [13,14,32,33], and that it could be an indicator for an aggressive disease course for OV [14]. A recent study demonstrates that KIFC1 is involved in the epithelial-to-mesenchymal transition and in the metastasis of LIHC via gankyrin/AKT signaling [34]. KIFC1 can be activated by transcription factor 4, and it can function as an oncogene to promote LIHC pathogenesis by regulating high-mobility-group AT-hook 1 transcriptional activity [13]. The present study also shows that the *KIFC1* expression was significantly increased in most of the tumors in the TCGA database, and that it is related to a poor prognosis (e.g., in ACC, KIRC, KIRP, LGG, LIHC, MESO, and SARC). These data suggest that the expression of KIFC1 could lead to carcinogenesis and to cancer metastasis in a variety of tumors, and that it warrants further investigation.

Among the top 100 genes that have similar expression patterns of *KIFC1* in the tumors of the TCGA cohort, the *KIFC1* expression was significantly and positively correlated with *KIF2C*, *NCAPH*, *KIF4A*, *TPX2*, and *CDCA5* expression in all of the tumor types in the TCGA database. Although there are no physical interactions between *KIFC1* and these five genes, these genes are all involved in the “cell cycle” and in “DNA replication”. KIF2C, a member of the kinesin-13 protein family, is involved in spindle formation via the stabilization of chromatin-associated microtubules [35]. A recent study has shown that KIF2C acts as a key factor in mediating the crosstalk between the Wnt-β-catenin and mTORC1 signaling in the pathogenesis of LIHC [36]. Although NCAPH plays a central role in mitotic chromosome assembly and segregation in humans [37], there is not much data on the relationship between NCAPH and cancer in the literature. TPX2 is also involved in spindle assembly, and it has been proposed as a marker for the diagnosis and prognosis of malignancies [38]. CDCA5 is essential for embryonic development, sister chromatid cohesion maintenance, and chromosome segregation [39]. A clinical study reports that sororin, which is encoded by *CDCA5*, is highly correlated to various proliferation markers in BRCA [40].

Using the data of both the KIFC1-interacted proteins and the *KIFC1*-correlated genes, the KEGG pathway analysis shows that targeting the cell cycle might be an important mechanism for the effect of *KIFC1* on carcinogenesis. The GO enrichment analysis further suggests that the genes that are directly interacted with or related to KIFC1 were mainly related to cell division and chromosome segregation. The GSEAs for the hallmark gene sets demonstrate that the high expression of *KIFC1* was associated with the genes for mitotic spindle assembly in various tumors of the TCGA cohort. One of the biological features for cancer is that, unlike normal cells, cancer cells divide continuously and excessively. Cells rely on cell cycle checkpoints to prevent the accumulation and propagation of genetic errors during cell division. Cancer cells typically stay in the metaphase three to five times longer than normal cells, which is very likely due to a sustained checkpoint activation, and, thus, a subsequently delayed mitotic exit [41]. KIFC1 frequently localizes between the microtubules within the metaphase spindle to bridge the microtubule cross-linking and to promote spindle bipolarity [3]. The depletion of KIFC1 leads to a dramatic increase in the multipolar anaphases, and it selectively induces cancer cell death in the cells with centrosome amplification [21]. Thus, KIFC1-induced centrosome amplification may lead to a delay of the mitotic exit in cancer. In the present study, the intersection analysis of the KIFC1-interacted proteins and the *KIFC1*-correlated genes identified two genes, *ASPM* and *TUBB,* as the potential important regulatory molecules that are associated with *KIFC1* for cancer cell division. Indeed, the interaction between KIFC1 and ASPM has been detected with a pull-down assay [42]. ASPM has been shown to participate in spindle organization, spindle orientation, and cytokinesis. It regulates the cell division by tuning cyclin E ubiquitination [43], and it is involved in the microtubule minus-end regulation at the spindle poles [42]. A clinical study has demonstrated that ASPM enhances the aggressiveness of PAAD by maintaining Wnt-β-catenin signaling [44]. A pull-down assay also reveals the interaction between KIFC1 and TUBB [45]. It is well known that TUBB acts as a major structural component of the microtubules that are critical for the cell division, shaping, motility, and the intracellular transport [46]. It has been shown that TUBB is associated with tumor aggressiveness and resistance to chemotherapy [47]. However, it is unclear whether the interactions among KIFC1, ASPM, and TUBB have synergistic effects on the progression of cancer. In this study, the GSEA shows that the high expression of *KIFC1* was associated with the oncogenic signatures, such as E2F and MYC, while the low expression of *KIFC1* was associated with the tumor suppression signature, p53. Of note, the roles of E2F, MYC, and p53 in cancer have been extensively studied [48,49,50].

Drug resistance continues to be one of the principal limiting factors to achieving favorable outcomes in patients with cancer. Clinical studies have shown that KIFC1 is associated with docetaxel resistance in PRAD and BRCA [18,19], with cisplatin resistance in BLCA [51], and with temozolomide resistance in GBM [52]. A recent study demonstrates that the ATM and ATR kinases phosphorylate KIFC1 to maintain the viability of cancer cells with centrosome amplification, which leads to drug resistance and tumor recurrence [20]. In the present study, we observed that a significant and positive correlation between the expression of *KIFC1* and the infiltration of MDSCs was present in all of the tumor types, except for HNSC-HPV+ and THCA, while the expression of *KIFC1* was negatively correlated with the ImmuneScore, which was used to quantify the in situ T-cell infiltration in most of the tumors in the TCGA database. MDSCs are immune-modulatory cells that suppress the adaptive immune responses and promote tumor progression and metastasis, and that are involved in multidrug resistance [53,54]. It has been reported that MDSCs produce eotaxin-1 to promote LUSC metastasis via the activation of ERK and AKT signaling [55]. A clinical study suggests that patients with STAD have higher levels of circulating MDSCs than healthy individuals, as well as higher levels of MDSCs that were correlated with the advanced cancer stage and reduced survival [56]. Another clinical study also shows that increased levels of MDSCs in patients with recurrent GBM are associated with poor prognoses [57]. These data support the hypothesis that the overexpression of KIFC1 in cancer cells may recruit more MDSCs, and not T cells, to infiltrate the tumor microenvironment, which thus leads to a poor prognosis.

Immunotherapy is an evolving cancer treatment that helps the immune system fight against cancer. Among the most promising approaches to activating therapeutic antitumor immunity is to block the immune checkpoints [58]. *KIFC1* is positively correlated with most of the immune checkpoint genes in the tumors of the TCGA cohort, especially *CD276*, *LAG3*, and *PD1*. CD276 is highly expressed in a wide range of human cancers, and it plays an important role in the inhibition of the T-cell function [59]. LAG3 signaling negatively regulates T-helper-cell activation, proliferation, and cytokine secretion, and tumor cells may use this signaling pathway to escape the immune surveillance [60]. PD1 is an inhibitory receptor that is expressed in all T cells during activation. Thus, the inhibition of PD1 enables an effective immune response against cancer cells, and it could be an important target for immuno-oncology therapy [61]. Unfortunately, many patients with positive initial responses could later develop resistance to the immune checkpoint inhibitors [62]. Of note, MDSCs could blunt the anticancer activity of the immune checkpoint inhibitors [63]. Both tumor MSIs and TMBs are promising predictive biomarkers for the efficacy of immune checkpoint inhibitors in cancer treatment. The finding of the present study suggests that *KIFC1* expression is significantly and positively correlated with the MSIs and TMBs in a large portion of the tumors in TCGA. The fact that KIFC1 plays an essential role in the centrosome amplification in cancer cells, and not in normal diploid cell division [21], suggests that KIFC1 may be an attractive therapeutic target for human cancers. Indeed, AZ82 is a small molecular inhibitor of KIFC1, and it causes centrosome declustering in cancer cells [64]. A combination of the small molecular inhibitors for KIFC1 and the immune checkpoint blocker(s) may be an effective option for overcoming the drug resistance in cancers. 

In conclusion, the present pan-cancer analyses of KIFC1 elucidates that the KIFC1 expression was correlated with the oncogenic signature gene sets, the MDSC infiltration, the ImmunoScore, the immune checkpoints, the MSI, the TMB, and the clinical prognosis across multiple tumors. These data may aid in the understanding of the role of KIFC1 in carcinogenesis and immunotherapy.

## Data Availability

Not applicable.

## Supplementary Materials

The following supporting information can be downloaded at: https://www.mdpi.com/article/10.3390/biomedicines10030637/s1. Figure S1. KIFC1 RNA expression level in different normal tissues using the consensus datasets of HPA and GTEx. nTPM, normalized transcripts per million. Figure S2. The KIFC1 expression level of different cancers or specific subtypes in TCGA database, Log2 TPM transformed expression data for plotting. N and T, normal and tumor tissue. Figure S3. Expression status of KIFC1 in various pathological stages of BRCA, KICH, KIRC, KIRP, LIHC, and LUAD in TCGA database. Log2(TPM + 1) transformed expression data for plotting, in one-way ANOVA. TPM, transcripts per million. Figure S4. Correlation analysis between KIFC1 expression and immune infiltration of cancer-associated fibroblasts, in purity-adjusted Spearman’s rho, with EPIC, MCPCOUNTER, XCELL, and TIDE algorithm. TPM, transcripts per million. Table S1. TCGA study abbreviations. Table S2. KIFC1 correlated and interacted genes based on String, and GEPIA2. Table S3. Raw data of correlation of ImmuneScore, StromalScore, and ESTIMATEScore with KIFC1 expression in tumors of TCGA database.
