# Supplementary material for: An Integrative Pan-Cancer Analysis of Kinesin Family Member C1 (KIFC1) in Human Tumors
Source: Biomedicines. 2022 Mar 10;10(3):637. doi: 10.3390/biomedicines10030637 (PMC8945479; doi:10.3390/biomedicines10030637)
Supplement: Supplementary file 1 [file biomedicines-10-00637-s001.zip › biomedicines-1610408-supplementary.pdf]

## Supplementary material

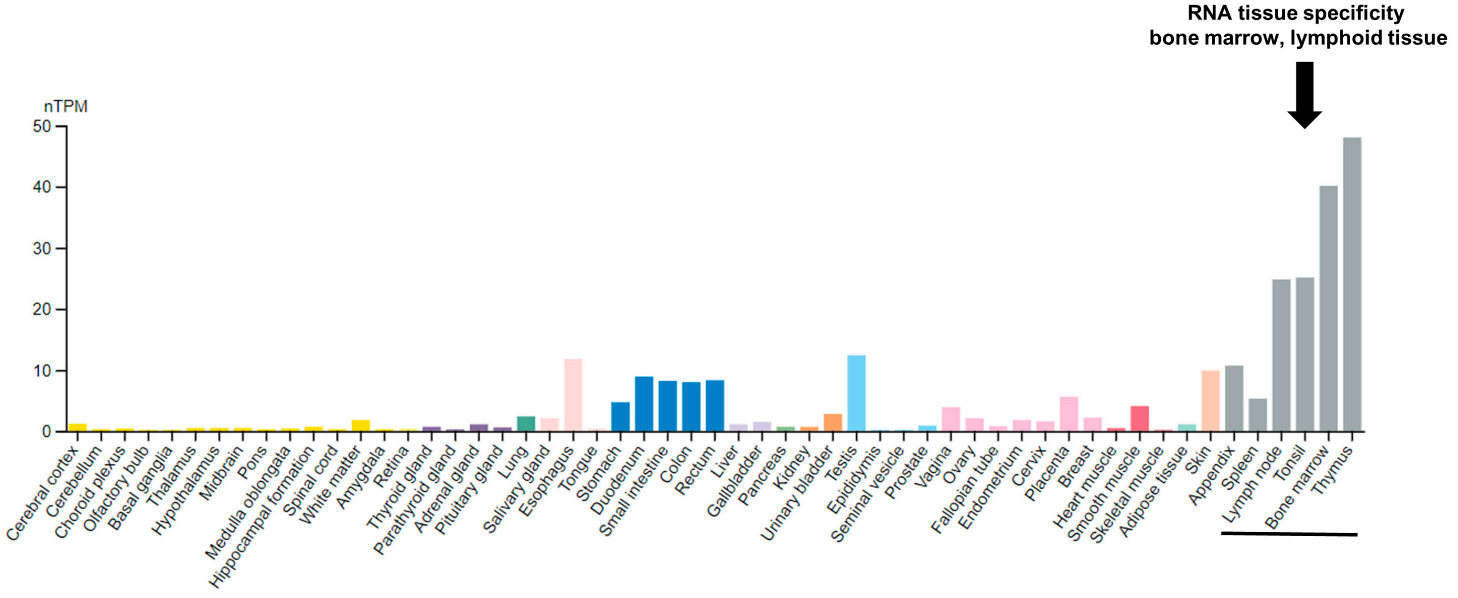


**Figure S1.** *KIFC1* RNA expression level in different normal tissues using the consensus datasets of HPA and GTEx. nTPM, normalized transcripts per million.

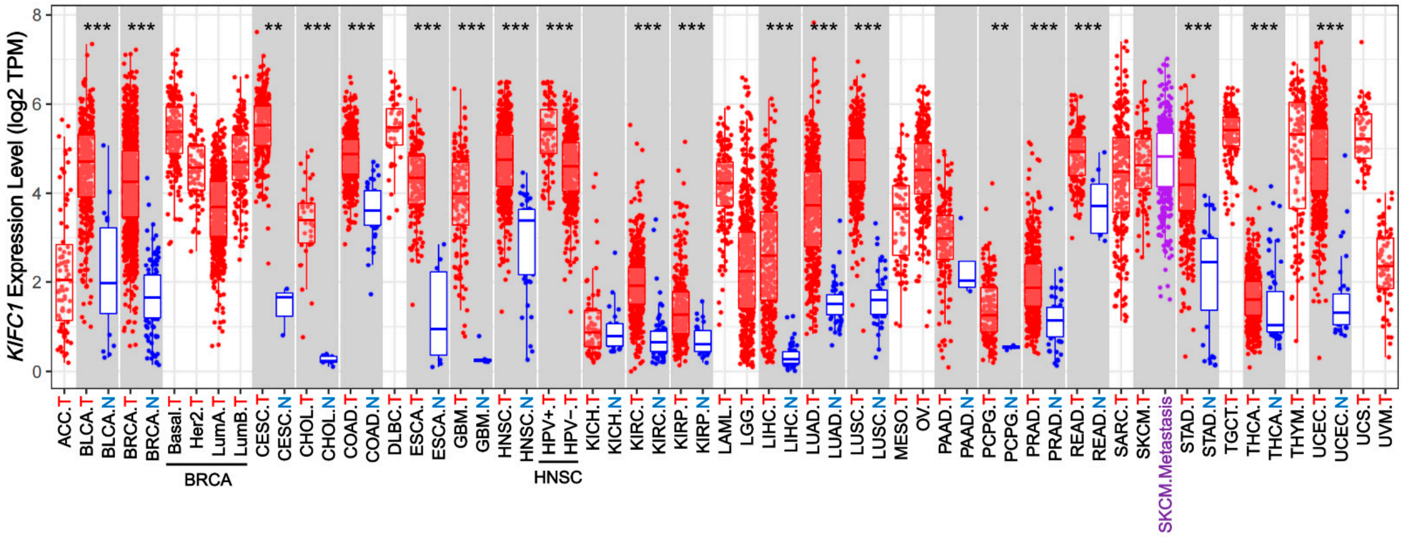


**Figure S2**. The *KIFC1* expression level of different cancers or specific subtypes in TCGA database, Log2 TPM transformed expression data for plotting. N and T, normal and tumor tissue.

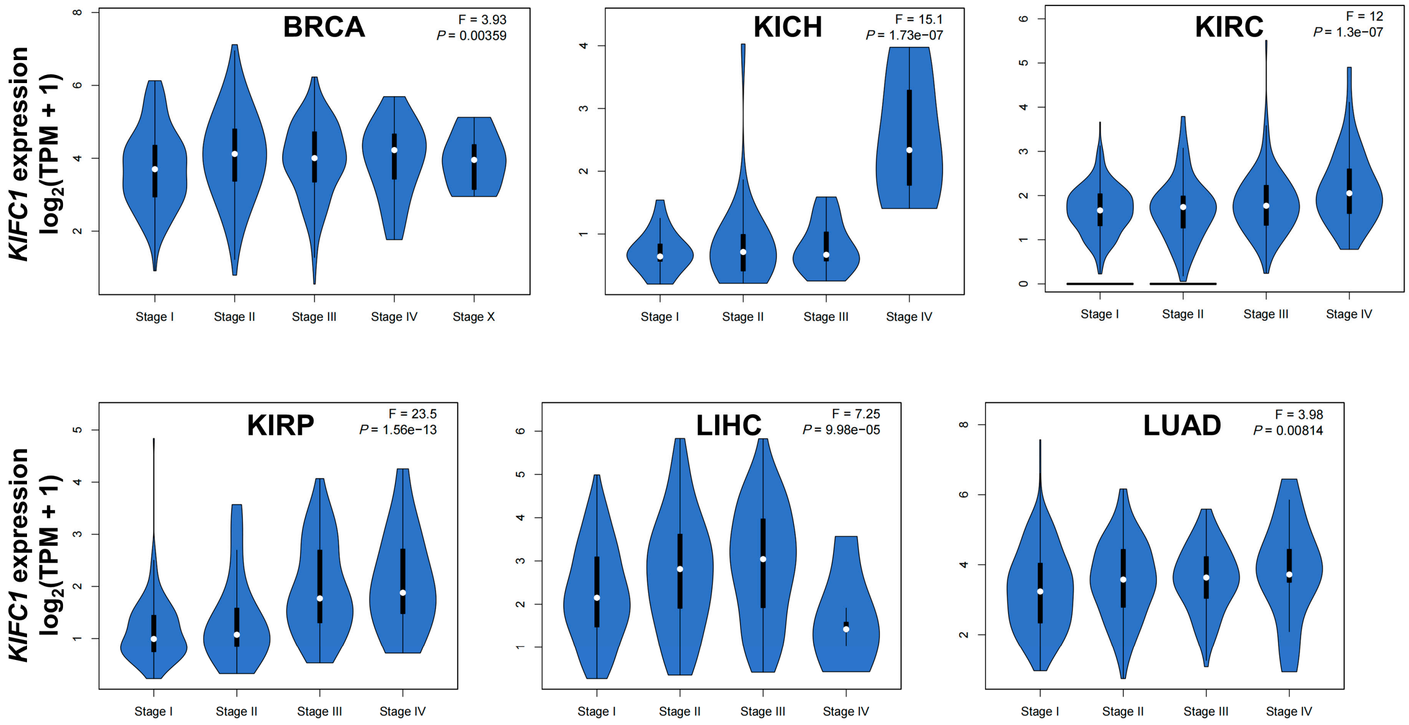


**Figure S3.** Expression status of KIFC1 in various pathological stages of BRCA, KICH, KIRC, KIRP, LIHC, and LUAD in TCGA database. $Log_2(TPM + 1)$ transformed expression data for plotting, in one-way ANOVA. TPM, transcripts per million.

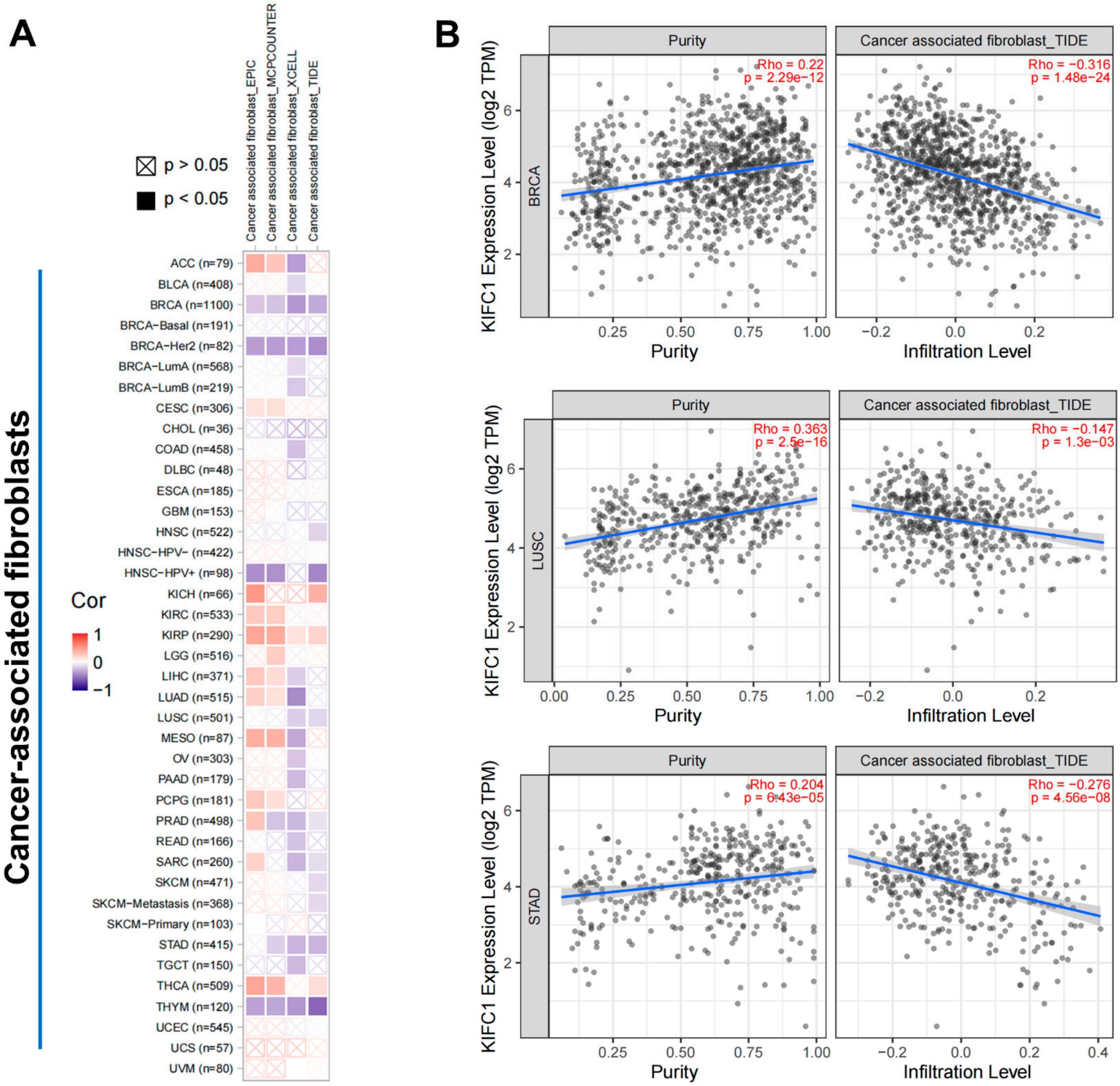


**Figure S4.** Correlation analysis between *KIFC1* expression and immune infiltration of cancer-associated fibroblasts, in purity-adjusted Spearman's rho, with EPIC, MCPCOUNTER, XCELL, and TIDE algorithm. TPM, transcripts per million.

| Abbreviations and acronyms | Full name |
| --- | --- |
| LAML | Acute Myeloid Leukemia |
| ACC | Adrenocortical carcinoma |
| BLCA | Bladder Urothelial Carcinoma |
| LGG | Brain Lower Grade Glioma |
| BRCA | Breast invasive carcinoma |
| CESC | Cervical squamous cell carcinoma and endocervical adenocarcinoma |
| CHOL | Cholangiocarcinoma |
| COAD | Colon adenocarcinoma |
| ESCA | Esophageal carcinoma |
| GBM | Glioblastoma multiforme |
| HNSC | Head and Neck squamous cell carcinoma |
| KICH | Kidney Chromophobe |
| KIRC | Kidney renal clear cell carcinoma |
| KIRP | Kidney renal papillary cell carcinoma |
| LIHC | Liver hepatocellular carcinoma |
| LUAD | Lung adenocarcinoma |
| LUSC | Lung squamous cell carcinoma |
| DLBC | Lymphoid Neoplasm Diffuse Large B-cell Lymphoma |
| MESO | Mesothelioma |
| OV | Ovarian serous cystadenocarcinoma |
| PAAD | Pancreatic adenocarcinoma |
| PCPG | Pheochromocytoma and Paraganglioma |
| PRAD | Prostate adenocarcinoma |
| READ | Rectum adenocarcinoma |
| SARC | Sarcoma |
| SKCM | Skin Cutaneous Melanoma |
| STAD | Stomach adenocarcinoma |
| TGCT | Testicular Germ Cell Tumors |
| THYM | Thymoma |
| THCA | Thyroid carcinoma |
| UCS | Uterine Carcinosarcoma |
| UCEC | Uterine Corpus Endometrial Carcinoma |
| UVM | Uveal Melanoma |

**Table S1. TCGA study abbreviations.**

| Correlated genes | Interacted genes |
|---|---|
| KIF2C | ASPM |
| NCAPH | BAG1 |
| KIF4A | BRCA1 |
| TPX2 | CCDC8 |
| CDCA5 | CDC34 |
| AURKB | CETN1 |
| GTSE1 | CETN2 |
| KIF15 | CXXC1 |
| CCNB2 | ENSP00000451560 |
| NUSAP1 | FAM188A |
| NCAPG | HTRA4 |
| CENPF | MAPRE1 |
| ZWINT | MAPRE2 |
| PRC1 | MAPRE3 |
| CDC25C | MAPT |
| KIF20A | MPG |
| RAD54L | NKRF |
| CCNB1 | NUP50 |
| KIF11 | NUP98 |
| FEN1 | PLEKHA4 |
| HJURP | PLEKHM1 |
| KIF23 | RNF169 |
| TTK | SET |
| DLGAP5 | STK10 |
| BIRC5 | TRMT10C |
| EXO1 | TUBA1A |
| MCM3 | TUBA1B |
| PLK1 | TUBA1C |
| NDC80 | TUBA3C |
| NUF2 | TUBA3D |
| BUB1 | TUBA3E |
| LMNB1 | TUBA4A |
| CENPA | TUBA8 |
| CDC20 | TUBAL3 |
| TROAP | TUBB |
| CDCA3 | TUBB1 |
| SPC25 | TUBB2A |
| SKA1 | TUBB2B |
| ASPM | TUBB3 |
| UBE2T | TUBB4A |
| PLK4 | TUBB4B |
| MCM6 | TUBB6 |
| CDK1 | TUBB8 |
| TUBB | TUBB8B |
| CCNA2 | TUBD1 |
| EZH2 | TUBE1 |
| CCNF | TUBG1 |

POC1A
TRAIP
KIF18B
RACGAP1
SGOL1
MELK
TIMELESS
NEK2
CDCA8
C16orf59
CDC45
PCNA
GINS1
TCF19
MKI67
UBE2C
ASF1B
CENPI
NCAPG2
FBXO5
ESPL1
OIP5
FANCI
CHEK1
CKAP2L
CHAF1A
MCM2
CENPO
RNASEH2A
KPNA2
FOXM1
KIF14
MND1
H2AFV
CHAF1B
MAD2L1
CDKN3
MCM10
SGOL2
DNAJC9
ORC6
C17orf53
LRR1
KIF18A
LMNB2
HMGB2
CEP55
ALYREF
TUBG2
UBXN7
WWP2

DTL
CDT1
MIS18A
E2F1
MTFR2

**Table S2.** *KIFC1* correlated and interacted genes based on String, and GEPIA2.

| Method | StromalScore pearson_R | StromalScore pearson_P | ImmuneScore pearson_R | ImmuneScore pearson_P | ESTIMATEScore pearson_R | ESTIMATEScore pearson_P |
|---|---|---|---|---|---|---|
| TCGA-GBM(N=152) | -0.462690217 | 1.96E-09 | -0.491492875 | 1.28E-10 | -0.49754214 | 6.98E-11 |
| TCGA-LGG(N=504) | -0.019601599 | 0.660658396 | 0.032574365 | 0.465591593 | 0.012930828 | 0.772131707 |
| TCGA-BRCA(N=1077) | -0.331029141 | 5.89E-29 | -0.028762632 | 0.345669764 | -0.184319585 | 1.10E-09 |
| TCGA-UCEC(N=178) | -0.303942491 | 3.71E-05 | -0.267379693 | 0.000308478 | -0.308590795 | 2.78E-05 |
| TCGA-CESC(N=291) | -0.254779123 | 1.08E-05 | -0.310272293 | 6.52E-08 | -0.322425639 | 1.83E-08 |
| TCGA-LUAD(N=500) | -0.210781118 | 1.99E-06 | -0.202045497 | 5.27E-06 | -0.222099882 | 5.26E-07 |
| TCGA-ESCA(N=181) | -0.248573282 | 0.000740413 | -0.339665166 | 2.90E-06 | -0.320053888 | 1.12E-05 |
| TCGA-SARC(N=258) | -0.242606816 | 8.25E-05 | -0.184522717 | 0.002929212 | -0.2223943 | 0.000318188 |
| TCGA-KIRP(N=285) | 0.05994894 | 0.313207722 | -0.000460625 | 0.99382278 | 0.024826348 | 0.676430197 |
| TCGA-COAD(N=282) | -0.211875019 | 0.000339742 | -0.108747064 | 0.068231656 | -0.17282001 | 0.003600585 |
| TCGA-PRAD(N=495) | -0.003676337 | 0.934975284 | 0.047303464 | 0.293551278 | 0.026691242 | 0.553551405 |
| TCGA-STAD(N=388) | -0.393195643 | 8.52E-16 | -0.258087172 | 2.54E-07 | -0.354871566 | 5.87E-13 |
| TCGA-HNSC(N=517) | -0.231247664 | 1.05E-07 | -0.025693319 | 0.55996906 | -0.13837302 | 0.001611352 |
| TCGA-KIRC(N=528) | 0.204468428 | 2.17E-06 | 0.307473896 | 5.04E-13 | 0.295866262 | 3.97E-12 |
| TCGA-LUSC(N=491) | -0.405669049 | 7.11E-21 | -0.372660766 | 1.27E-17 | -0.41135674 | 1.79E-21 |
| TCGA-LIHC(N=363) | -0.273800325 | 1.16E-07 | -0.01211605 | 0.818049793 | -0.134442711 | 0.010338568 |
| TCGA-SKCM(N=452) | -0.198708196 | 2.09E-05 | -0.140513389 | 0.002753906 | -0.173974974 | 0.000201683 |
| TCGA-BLCA(N=405) | -0.064070246 | 0.198189829 | 0.023849337 | 0.632263588 | -0.02124507 | 0.669907161 |
| TCGA-THCA(N=503) | 0.278178386 | 2.17E-10 | 0.359629453 | 8.38E-17 | 0.351323598 | 4.66E-16 |
| TCGA-MESO(N=85) | 0.063684588 | 0.562563733 | -0.048075384 | 0.662167005 | -0.003488223 | 0.974724254 |
| TCGA-READ(N=91) | -0.16843286 | 0.110497643 | -0.124620176 | 0.239210887 | -0.157385808 | 0.136247204 |
| TCGA-OV(N=417) | -0.14701273 | 0.002617289 | -0.103186414 | 0.035167295 | -0.134477134 | 0.005952966 |
| TCGA-UVM(N=79) | -0.084126828 | 0.461046074 | -0.042531666 | 0.709764267 | -0.05818832 | 0.61048768 |
| TCGA-PAAD(N=177) | -0.010449115 | 0.89021257 | 0.061357051 | 0.417202853 | 0.027438989 | 0.716953159 |
| TCGA-TGCT(N=132) | -0.309146724 | 0.000309984 | -0.376702794 | 8.51E-06 | -0.432089222 | 2.30E-07 |
| TCGA-UCS(N=56) | -0.111170129 | 0.414677193 | -0.291130556 | 0.029489681 | -0.235802341 | 0.080196336 |
| TCGA-LAML(N=214) | 0.317651198 | 2.11E-06 | -0.072097514 | 0.293770798 | 0.108596528 | 0.113190539 |
| TCGA-PCPG(N=177) | -0.034449182 | 0.64896216 | -0.108685397 | 0.149870389 | -0.075033398 | 0.32091389 |
| TCGA-ACC(N=77) | -0.139983034 | 0.224654594 | -0.164342101 | 0.153222856 | -0.16070539 | 0.162650685 |
| TCGA-DLBC(N=46) | 0.065487737 | 0.665450262 | -0.112250356 | 0.457651742 | -0.032393736 | 0.83076962 |
| TCGA-KICH(N=65) | 0.069916448 | 0.579972358 | -0.043541388 | 0.730550045 | 0.006106558 | 0.961494844 |
| TCGA-CHOL(N=36) | -0.303780247 | 0.071672798 | -0.163552366 | 0.340528918 | -0.228579234 | 0.179951152 |

**Table S3.** Raw data of correlation of ImmuneScore, StromalScore, and ESTIMATEScore with *KIFC1* expression in tumors of TCGA database.
